# Supplementary material for: Can Blood Ammonia Level, Prehospital Time, and Return of Spontaneous Circulation Predict Neurological Outcomes of Out-of-Hospital Cardiac Arrest Patients? A Nationwide, Retrospective Cohort Study
Source: J Clin Med. 2022 May 4;11(9):2566. doi: 10.3390/jcm11092566 (PMC9105173; doi:10.3390/jcm11092566)
Supplement: Supplementary file 1 [file jcm-11-02566-s001.zip › jcm-1690399-supplementary.pdf]

Table S1. Characteristics of patients in the cardiac causes group and the non-cardiac causes group at hospital arrival.

	All participants (n=7426)	Cardiac arrest patients (n=3719)	Non-cardiac arrest patients (n=3707)	*p*-value
Age (year), median (IQR)	76 (64–84)	76 (65–84)	76 (63–85)	<0.01
Male, n (%)	4538 (61%)	2428 (65%)	2110 (57%)	<0.01
Total prehospital time* (min), median (IQR)	34 (28–41)	33 (27–41)	34 (29–42)	<0.01
Witnessed collapse, n (%)	3181 (47%)	1578 (42%)	1603 (43%)	0.46
Bystander CPR, n (%)	3202 (47%)	1647 (44%)	1555 (42%)	0.035
AED used by bystander, n (%)	149 (2.2%)	130 (3.8%)	19 (0.56%)	<0.01
Adrenalin used by EMS	2278 (31%)	1094 (32%)	1184 (35%)	0.016
Initial rhythm at hospital arrival				
VF/pulseless VT, n (%)	392 (5.3%)	329 (9.0%)	63 (2.0%)	<0.01
PEA/asystole, n (%)	6124 (82%)	2964 (80%)	3160 (85%)	<0.01
ROSC during transport, n (%)	910 (12%)	426 (11%)	484 (13%)	<0.01
Blood ammonia level (μg/dL), median (IQR)	253 (125–438)	251(120–426)	254 (130–454)	<0.01
Potassium (mEq/L), median (IQR)	6.0 (4.6–7.9)	5.9 (4.4–7.8)	6.1 (4.7–8)	<0.01
Lactate (mg/dL), median (IQR)	118 (74–156)	120 (77–156)	116 (72–156)	0.023
TTM, n (%)	634 (8.5%)	490 (13%)	144 (4%)	<0.01
Patients admitted to the hospital, n (%)	2457 (33%)	1194 (32%)	1263 (34%)	0.072
Survival at 30 days, n (%)	738 (10%)	489 (13%)	249 (7%)	<0.01
Favorable neurological outcome at 30 days, n (%)	364 (4.9%)	302 (8.1%)	62 (1.7%)	<0.01

Abbreviations: interquartile range (IQR); cardiopulmonary resuscitation (CPR); automated external defibrillator (AED); emergency medical services (EMS); ventricular fibrillation (VF); ventricular tachycardia (VT); pulseless electrical activity (PEA); return of spontaneous circulation (ROSC); targeted temperature management (TTM). * Total prehospital time was defined as the time span from awareness to hospital arrival.

Table S2. Univariable and multivariable logistic regression analysis of blood ammonia levels for favorable neurological outcome based upon whether the causes of cardiac arrest were cardiac or non-cardiac.

	Crude OR (95% CI)	Adjusted OR (95% CI)
Cardiac cause	0.988 (0.986–0.989)	0.996 (0.994–0.999)
Non-cardiac causes	0.991 (0. 988–0.994)	0.995 (0.990–0.999)

Abbreviations: odds ratio (OR); confidence interval (CI). Adjustments for multivariable logistic regression: age, sex, witnessed collapse, bystander cardiopulmonary resuscitation, automated external defibrillator use on scene, adrenaline use on scene, electrocardiogram on arrival, and potassium, lactate, and ammonia levels at hospital arrival.

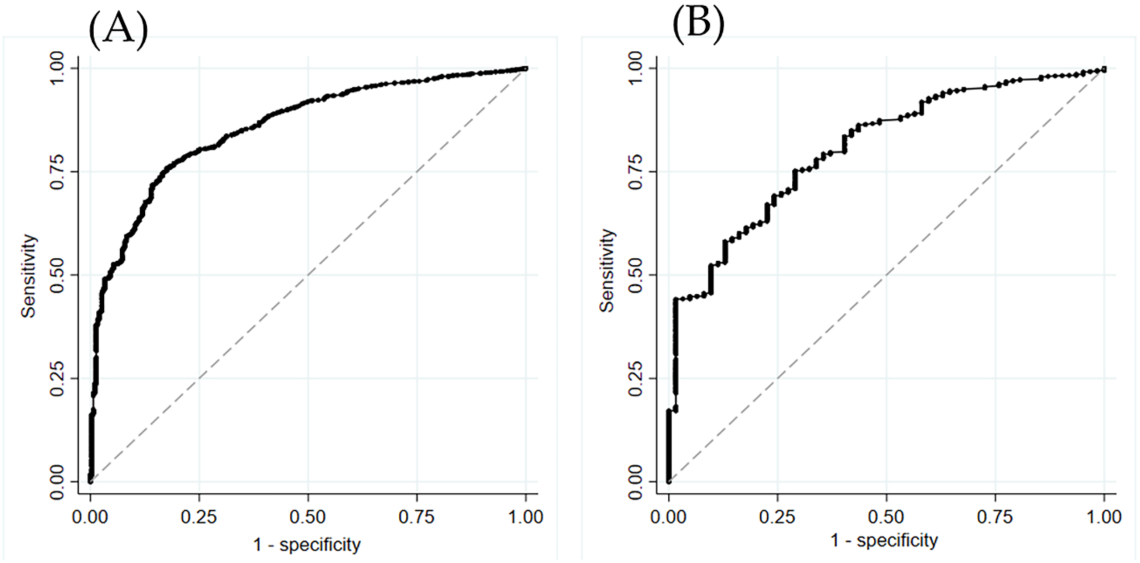

Figure S1. ROC curve for predicting poor outcomes with blood ammonia levels in the cardiac causes group (**A**) and the noncardiac causes group (**B**). The areas under the ROC curve for the cardiac causes group and the noncardiac causes group for the prediction of poor outcomes were 0.856 (95% CI: 0.836–0.875) and 0.805 (95% CI: 0.757–0.853), respectively. Abbreviations: receiver operating characteristic (ROC).

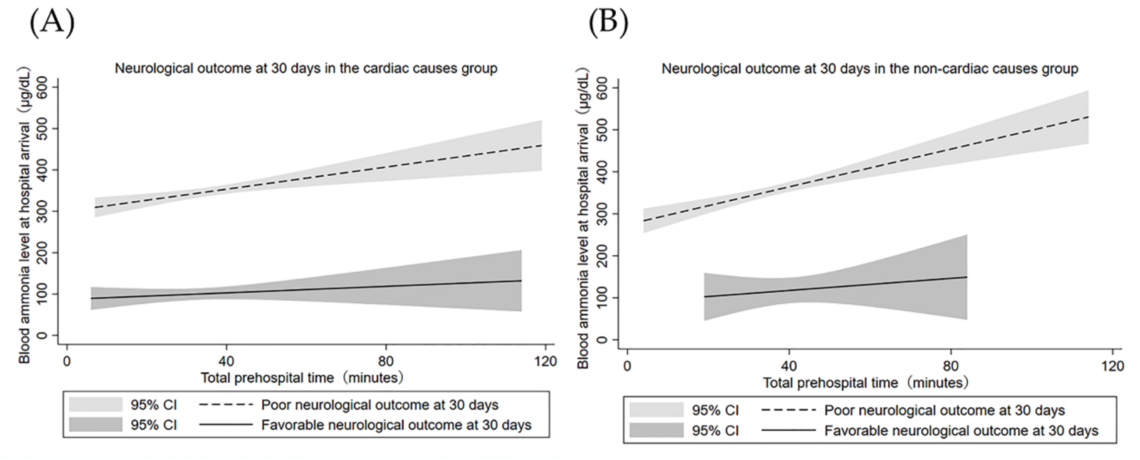

Figure S2. Relationship between blood ammonia levels and total prehospital time in the cardiac causes group (**A**) and the noncardiac causes group (**B**). Blood ammonia levels at hospital arrival increased with total prehospital time. Blood ammonia levels at hospital arrival were a reliable predictor in both the cardiac causes group and the noncardiac causes group. Blood ammonia level measured at hospital arrival more precisely predicted neurological outcomes in the cardiac causes group compared to the noncardiac causes group. Abbreviation: confidence interval (CI).
